# HbA1c and the Risk of Lower Limb Ulcers Among Diabetic Patients: An Observational and Genetics Study

**DOI:** 10.1155/jdr/4744194

**Published:** 2025-03-29

**Authors:** Guojun Guo, Yunlong Guan, Yuhuan Chen, Yuge Ye, Zeyu Gan, Xi Cao, Zhenbing Chen, Xingjie Hao

**Affiliations:** ^1^Department of Hand Surgery, Union Hospital, Tongji Medical College, Huazhong University of Science and Technology, Wuhan, China; ^2^Department of Epidemiology and Biostatistics, School of Public Health, Tongji Medical College, Huazhong University of Science and Technology, Wuhan, Hubei, China; ^3^School of Medicine and Health Management, Tongji Medical College, Huazhong University of Science and Technology, Wuhan, Hubei, China

**Keywords:** cohort study, diabetes, HbA1c, lower limb ulcer, Mendelian randomization

## Abstract

**Aims:** A lower limb ulcer is a serious complication of diabetes. The association between glycated hemoglobin (HbA1c) and lower limb ulcers needs further clarification. We aimed to comprehensively evaluate the relationship between HbA1c and lower limb ulcer risk in diabetic patients through multivariable-adjusted observational analyses and Mendelian randomization (MR) approaches.

**Methods:** This prospective cohort study included 23,434 individuals with prevalent diabetes in the UK Biobank. The Cox proportional hazard model was used to evaluate the association between HbA1c levels and lower limb ulcer risk. Furthermore, a one-sample MR analysis was conducted to explore the potential causal effect.

**Results:**Over a follow-up period of 290,677 person-years (median length: 13.3 years), 1101 lower limb ulcers cases were documented. The multivariable-adjusted hazard ratios across categories of HbA1c of ≤ 42, 42–53, 53–64, 64–75, 75–86, and > 86 mmol/mol were 0.93 (0.76–1.15), 1.00, 1.24 (1.05–1.46), 1.98 (1.65–2.39), 2.68 (2.13–3.37), and 4.52 (3.62–5.65), respectively. The restrictive cubic spline model showed that lower limb ulcer risk increased steeply when HbA1c concentration exceeded 53 mmol/mol. One-sample linear and nonlinear MR analyses provided a positive but not significant association between genetically proxied HbA1c and lower limb ulcer risk among diabetic patients.

**Conclusions:** High HbA1c levels increase the risk of lower limb ulcers in diabetic patients. MR analyses validated the positive but not significant association between genetically proxied HbA1c levels and lower limb ulcer risk. The results recommended an HbA1c goal of < 53 mmol/mol to decrease the incidence of diabetic ulcers.

## 1. Introduction

Lower limb ulcers, including leg ulcers and foot ulcers, are common and frequently occurring diseases, affecting approximately 1%–2% of US adults, leading to a massive financial burden on public health [1]. The most common etiologies of lower limb ulcers include diabetic foot ulcers (DFUs), venous leg ulcers (VLUs), arterial ulcers, and pressure ulcers [2]. Thereinto, DFU is the primary cause of nontraumatic lower extremity amputation (LEA) and a serious and disastrous complication of diabetes, typically manifested as ulcers, gangrene, infection, or tissue destruction [3]. Approximately 529 million people worldwide have diabetes in 2021 [4], while an estimated 9.1–26.1 million diabetic patients worldwide eventually develop foot ulcers each year [5]. In particular, DFUs account for approximately 80% of foot ulcers [6], and persons with diabetes have a lifetime risk of 19%–34% for developing DFU [5]. Thus, based on the high incidence rate of lower limb ulcers in diabetes, it is necessary to investigate the risk factors for lower limb ulcers.

Effective management and early detection can prevent and reduce the severity of diabetic complications, including foot ulcers. Identifying the high-risk foot is a crucial part of diabetic therapy. Risk factors for DFU include both patient- and foot-specific factors, such as age, glycemic management, smoking, cardiovascular disease, chronic kidney disease, and retinopathy [7]. Prior studies have reported that DFUs are more common among males than among females, and DFU patients are older, have a longer diabetic duration, and have a history of smoking than patients without DFUs [8, 9]. Additionally, a systematic review showed that physical activity and exercise could effectively lower the risk of DFUs [10]. However, there is conflicting evidence regarding the association between obesity and risk of DFUs. Some studies revealed that overweight and obesity are risk factors associated with DFUs [11, 12], while others indicated that a lower body mass index (BMI) is a risk factor for amputation in DFU patients [13, 14] or BMI has no significant association with DFUs [15]. In addition, one study showed that there is a J-shaped correlation between BMI and DFUs, and patients with a BMI less than 25 kg/m^2^ or greater than 45 kg/m^2^ have a higher chance of getting DFUs [16].

Specifically, glycemic control is the most crucial metabolic factor in DFU patients [17]. Blood glucose optimization is widely advised to enhance wound healing and reduce harmful effects on infection and the inflammatory response, and glycemic control could reduce the risk of LEA [18]. Therefore, glycemic management is regarded as a fundamental component of DFU treatment [17]. Glycated hemoglobin (HbA1c) concentration is a valuable indicator of long-term glycemic control [19]. Notably, a chronically increased HbA1c level is an independent risk factor for DFU [7]. A previous study showed that the daily wound area healing rate dropped by 0.028 cm^2^ per day for every 1% increase in HbA1c, which could be an important predictor of wound healing in diabetes [20]. HbA1c levels ≥ 8% (equivalent to 64 mmol/mol) were associated with a higher risk of LEA in DFU patients [21]. However, other studies found no significant association between HbA1c levels and the risk of DFU [22, 23]. Besides, Xiang et al. suggested that slightly higher HbA1c levels of 7%–8% (53–64 mmol/mol) could facilitate ulcer healing in DFU patients during treatment [24]. Hence, despite these discrepancies, more research into the correlation between HbA1c levels and lower limb ulcers in diabetes is needed. Identifying an evidence-based, clinically actionable HbA1c threshold for primary prevention of ulcers remains an important issue in diabetes management.

The main purpose of the present study was to explore the relationship between HbA1c levels and the risk of lower limb ulcers in diabetic patients according to the large prospective cohort of the UK Biobank. Different HbA1c levels and their associations with lower limb ulcers were systematically evaluated, and the optimum HbA1c level to prevent lower limb ulcers in diabetes was further identified.

## 2. Methods

### 2.1. Study Population

The UK Biobank is a large prospective cohort study that recruited over 500,000 participants ranging from 40 to 69 years when recruited in 2006–2010 in England, Scotland, and Wales. Extensive data were obtained through touchscreen questionnaires, physical measurements, and biological samples at recruitment. Specific methods of data collection have been described previously [25]. All participants gave informed consent, and the study was approved by the North West–Haydock Research Ethics Committee (16/NW/0274).

**Figure 1:**
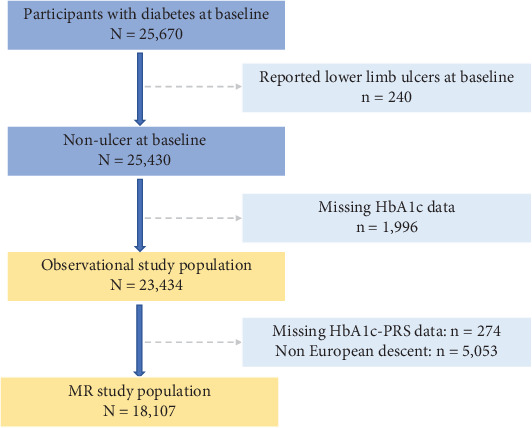
Flowchart of participants in this study.

There were 25,670 participants with diabetes diagnosis at baseline, including 1754 Type 1 diabetes (T1D) and 23,916 Type 2 diabetes (T2D). The diagnosis of prevalent diabetes was based on a validated algorithm, which utilized self-reported and nurse-interviewed medical history, medication history, and hospital inpatient records to identify diabetes [26]. After excluding participants with a history of lower limb ulcers (*n* = 240) or without an HbA1c measurement (*n* = 1996), a total of 23,434 participants were included in the observational study. In addition, 18,107 individuals of Caucasian ancestry with a valid genetic risk data for HbA1c were included in the Mendelian randomization (MR) analysis, as presented in Figure 1.

### 2.2. Measurement of HbA1c

Blood collection sampling procedures for the UK Biobank have previously been described and validated [27]. The HbA1c levels were measured by the VARIANT II TURBO hemoglobin testing system on a Bio-Rad and in mmol/mol units. The equation between the NGSP network (%HbA1c) and the IFCC network (mmol/mol) was NGSP = 0.0915∗IFCC + 2.15 [28].

### 2.3. Ascertainment of Lower Limb Ulcer Event

The UK Biobank mainly records disease through four channels: self-report medical history, linkage to hospital inpatient admissions, linkage to national death registries, and linkage to primary care data. The lower limb ulcer event was defined as L97 based on the *International Classification of Disease 10^th^ Revision* (*ICD-10*). The first occurrence time of L97 for participants was determined based on the terms of Data-Field 131834 in the UK Biobank.

### 2.4. Assessment of Covariates

To reduce the impact of confounding factors, demographics, socioeconomic status, lifestyle factors, and medical history were selected as covariates for the models, which included age, sex, BMI, Townsend deprivation index, ethnicity, smoking status, drinking status, physical activity, season of blood collection, and the duration of diabetes. Data were collected at baseline using a touchscreen questionnaire or body measurements. BMI was calculated as body weight in kilograms divided by the square of height in meters, which were measured by trained nurses at baseline. Season of blood collection was categorized according to the months in which participants attended the assessment centers. Socioeconomic deprivation was evaluated using Townsend deprivation index scores, with higher scores representing higher levels of socioeconomic deprivation [29]. Smoking status was categorized as never, previous, or current. Alcohol consumption was calculated based on the frequency of consumption on a typical day, week, or month. Physical activity was categorized as never, low, medium, or high, as previously described in prior literature [30]. The duration of diabetes was calculated as the date of recruitment minus the date of diabetes diagnosis.

### 2.5. Observational Analysis

Based on the lower limb ulcer status, the baseline characteristics of participants were described as median (interquartile range, IQR) for continuous variables and number (percentage) for categorical variables. As described in our previous study [31], missing values were imputed by the median value for continuous variables or were replaced as a missing indicator category for categorical variables. The Mann–Whitney *U* test or chi-square test was used to test the differences in baseline characteristics by lower limb ulcer status. Person-years for each participant were calculated from the date of entry into the UK Biobank to the date of diagnosis of lower limb ulcers, death, lost follow-up, or October 15, 2022, whichever was the earliest.

The Cox proportional hazards regression model was used to estimate the hazard ratio (HR) and 95% confidence interval (CI) for the association between HbA1c levels and risk of lower limb ulcers. The proportional hazards assumption was evaluated using Schoenfeld residuals. The participants were divided into six groups based on baseline HbA1c concentrations (≤ 42, 42–53, 53–64, 64–75, 75–86, and > 86 mmol/mol, with the 42–53 group as the reference). The trend test was carried out by taking the special integer values (1, 2, 3, 4, 5, and 6) as a continuous variable in the model. Univariate and multivariate Cox proportional hazards regression models were fitted to minimize the impact of confounding factors. In Model 1, the Cox regression model was not adjusted for covariates. In Model 2, we adjusted for age, sex, BMI, Townsend deprivation index, race, smoking status, drinking status, physical activity, and season of blood collection. In Model 3, we further adjusted for the duration of diabetes.

The Kaplan–Meier curve was plotted using the inverse-variance weighting method with adjustment for covariates to compare the cumulative risks of lower limb ulcers according to different HbA1c levels. The restricted cubic spline (RCS) model with four knots was used to investigate the dose–response relationship between HbA1c concentration and the risk of lower limb ulcers, using 53 mmol/mol as the reference point.

We also conducted subgroup analyses according to age (< 60 years, ≥ 60 years), sex (female, male), BMI (< 30 kg/m^2^, ≥ 30 kg/m^2^), smoking status (never, other), physical activity (never or low, medium or high), and diabetes duration (≤ 3 years, > 3 years). The likelihood ratio test was used to evaluate the interaction between HbA1c levels and grouping features. In addition, several sensitivity analyses were conducted to examine the robustness of the results. First, the analysis was restricted to the participants with complete covariates. Second, only participants with T2D at baseline were included. Third, participants were further adjusted for the use of diabetic medication. Fourth, we excluded participants with events during the first 2 years of follow-up. Fifth, to align the background factors, HbA1c groups were weighted using inverse probability weighting.

### 2.6. MR

To assess the causal effect of HbA1c on lower limb ulcers, we conducted one-sample linear and nonlinear MR analyses with the standard PRS of HbA1c derived from the UK Biobank (Data-Field 26238) as instrumental variables. As described previously, the standard PRS of HbA1c was calculated as the genome-wide sum of the per-variant effect size multiplied by allele dosage [32]. The correlation between the genetic instrument and HbA1c level was tested using linear regression. Linear or logistic regression between the genetic instrument and a series of risk factors was fitted to test the pleiotropy of the genetic instrument.

Linear MR analysis was based on the ratio method by dividing the genetic association with lower limb ulcers by the genetic association with HbAc1 concentration. For nonlinear MR analysis, the doubly ranked method was first used to estimate the homogeneity assumption that the effect of instrumental variables on exposure is linear and constant for all individuals in the population [33]. The doubly ranked method is a nonparametric method that was implemented by first ranking participants into prestrata according to their level of instrumental variables and second ranking participants within each prestratum into stratum according to their level of exposure [34]. After testing the homogeneity assumption, the residual method was used to estimate the shape of associations between genetically predicted HbAc1 and the risk of lower limb ulcers. The residual method was implemented by first regressing HbA1c on the standard PRS and second strata based on the residual from this regression [35]. All MR analyses were performed in the total population as well as in the male and female populations with adjustment for age, sex, genotype batch, and the first 10 genetic principal components.

All statistical analyses were conducted using R software (Version 4.3.1). A two-sided *p* value < 0.05 was considered statistically significant.

## 3. Results

### 3.1. Baseline Characteristics of Participants

The baseline characteristics of the 23,434 diabetes participants according to lower limb ulcer status are shown in Table 1. Over 290,677 person-years of follow-up (median length: 13.3 years), 1101 cases of lower limb ulcers were documented. The incidence density of lower limb ulcers was 3.79 per 1000 person-years. Participants who developed lower limb ulcers were more likely to be older, male, smokers, overweight, and obese and had a longer duration of diabetes and less physical activity. Table S1 summarizes the baseline characteristics according to HbA1c concentration. The average (standard deviation) of HbA1c concentration was 53.2 ± 13.9 mmol/mol, and the median (IQR) was 50.5 (43.7–59.6) mmol/mol. Participants with higher HbA1c concentrations were more likely to be younger, smokers, and nondrinkers; tended to have higher BMI and Townsend deprivation index; and had longer diabetes duration and less physical activity (Table S1).

### 3.2. Associations of HbA1c and the Risk of Lower Limb Ulcers

As shown in Table 2, we observed a significant positive association between HbA1c levels and the risk of lower limb ulcers in the crude model. For every 5.5 mmol/mol (equivalent to 0.5%) increase in HbA1c, the risk of lower limb ulcers increases by 19%. After adjusting for all covariates, higher HbA1c levels were still associated with an increased risk of lower limb ulcers (*p* for trend < 0.001). The multivariable-adjusted HRs across categories of HbA1c of Q1 (≤ 42 mmol/mol), Q2 (42–53 mmol/mol), Q3 (53–64 mmol/mol), Q4 (64–75 mmol/mol), Q5 (75–86 mmol/mol), and Q6 (> 86 mmol/mol) were 0.93 (95% CI: 0.76–1.15), 1.00 (reference), 1.24 (1.05–1.46), 1.98 (1.65–2.39), 2.68 (2.13–3.37), and 4.52 (3.62–5.65), respectively, for lower limb ulcers.

**Figure 2:**
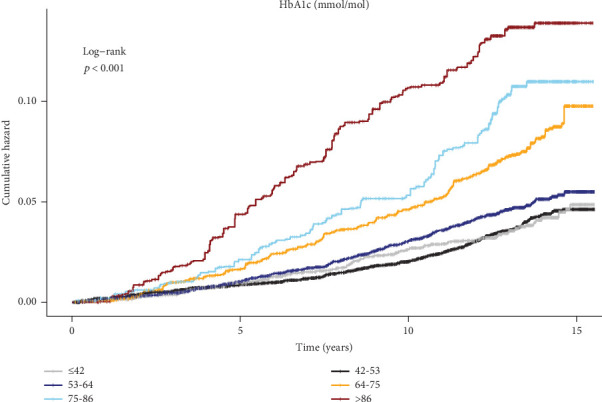
Kaplan–Meier curves for lower limb ulcers in patients with diabetes according to HbA1c level. The inverse-variance weighting method was used and adjusted for age, sex, BMI, ethnicity, Townsend deprivation index, smoking status, drinking status, physical activity, season of blood collection, and duration of diabetes.

**Figure 3:**
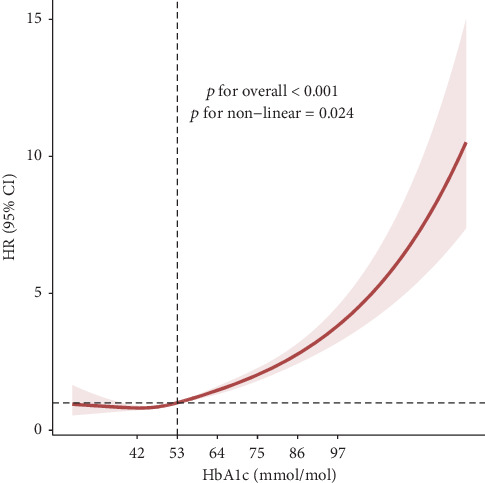
Exposure–response relationships of HbA1c with the risk of lower limb ulcers. HRs were adjusted for age, sex, BMI, Townsend deprivation index, ethnicity, smoking status, drinking status, physical activity, season of blood collection, and duration of diabetes.

The Kaplan–Meier curve illustrated significantly different cumulative risks of lower limb ulcers according to different HbA1c levels (log-rank *p* < 0.001), with higher HbA1c levels associated with higher cumulative risks (Figure 2). By establishing the RCS model adjusted for all covariates, we observed a nonlinear dose–response relationship between HbA1c and the risk of lower limb ulcers (*p* for overall < 0.001and *p* for nonlinear = 0.024). When the HbA1c concentration exceeded 53 mmol/mol, the risk of lower limb ulcers increased steeply with the increase in HbA1c, while further reduction of HbA1 did not significantly reduce the risk of lower limb ulcers when HbA1c < 53 mmol/mol (Figure 3).

### 3.3. Subgroup and Sensitivity Analyses

Stratified analyses were conducted based on several potential risk factors, including sex, age, BMI, smoking status, physical activity, and duration of diabetes. The association between HbA1c and the risk of lower limb ulcers was consistent in different subgroups (Table 3). There was no significant multiplicative interaction between the grouped variables and HbA1c level (Table S2). Besides, all the sensitivity analyses proved the robustness of the results, as listed in Tables S3–S7. After aligning background factors, the effect size of HbA1c on lower limb ulcers slightly decreased but remained statistically significant (Table S7).

### 3.4. MR

**Figure 4:**
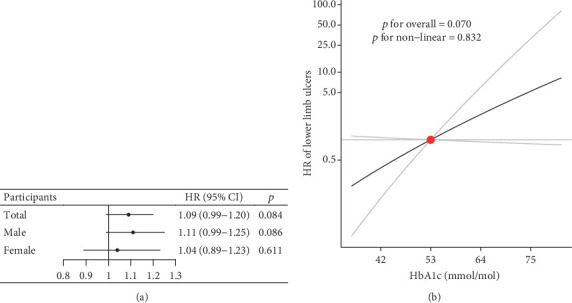
Linear and nonlinear MRs estimated for HbA1c on the risk of lower limb ulcers. (a) Linear MR estimated for both the total population and only male and only female population. (b) Nonlinear MR estimated fitted by the residual method with 53 mmol/mol as the reference for the total population. The grey lines represent the 95% CI.

The PRS for HbA1c was strongly associated with HbA1c concentrations, with an *F* statistic of 56.3, which was above the standard cutoff (> 10), indicating sufficient instrumental strength [36]. The genetic instrument was independent of risk factors, such as lipid profile and blood pressure, ruling out the risk of horizontal pleiotropy (Figure S1). In the linear MR analysis, the HR for incident lower limb ulcers was 1.09 (0.99–1.20, *p* = 0.084) for a 1-mmol/mol increase in HbA1c in total diabetic patients and 1.11 (0.99–1.25, *p* = 0.086) for male and 1.04 (0.89–1.23, *p* = 0.611) for female diabetic patients (Figure 4).

For nonlinear MR analysis, the doubly ranked method indicated that the effects of the genetic instrument on HbA1c were approximately consistent within each stratum, confirming the homogeneity assumption (Table S8). Therefore, the residual method was used for the main analysis. As shown in Figure 4, the residual method demonstrated a positive linear association between genetically proxied HbA1c and the risk of lower limb ulcers in total participants, but the association was not significant (*p* for overall = 0.070 and *p* for nonlinear = 0.832). A similar result was observed in the doubly ranked analysis (Figure S2). In sex-stratified analyses, we observed no statistical evidence favoring a significant association between genetically proxied HbA1c and the risk of lower limb ulcers in any of the analyses (Figure S3).

## 4. Discussion

In this prospective study of lower limb ulcers in diabetes, we discovered that a high HbA1c concentration was significantly associated with an increased risk of lower limb ulcers. In addition, both subgroup and sensitivity analyses supported this finding that HbA1c levels were positively associated with the risk of lower limb ulcers. MR analysis validated the positive but not significant association between genetically proxied HbA1c levels and the risk of lower limb ulcers. Our research offers insights into the primary prevention of lower limb ulcers in diabetes.

A lower limb ulcer is a long-term complication of diabetes, and the pathogenesis of diabetic ulcers is complex. Chronic hyperglycemia and insulin resistance can cause increased oxidative stress, endothelial damage, proinflammatory gene expression, and platelet activation via a variety of pathways such as the polyol and hexosamine pathways [37]. Subsequently, these pathophysiological alterations may result in neuropathy, peripheral arterial disease, inflammatory cytokines, and increased vulnerability to infection [37, 38]. Among them, diabetic neuropathy is frequently accompanied by loss of protective sensation in the foot, which makes the local tissue vulnerable to physical trauma [6]. Repetitive injury can lead to inflammation, tissue necrosis, and eventually ulceration [39]. Additionally, peripheral arterial disease can cause impaired wound healing due to decreased skin perfusion and contribute to the pathophysiology and chronicity of ulcers [40]. Consequently, chronic hyperglycemia is a major initial factor in the pathological process of ulceration in diabetes.

HbA1c is a specific type of glycated hemoglobin formed from the binding of glucose to the N-terminal valine of the hemoglobin *β*-chain [41]. This bond is reversible at first but progressively transforms into an irreversible stable form when keto-amino binding occurs. Blood glucose levels and erythrocyte life duration are two factors that affect HbA1c concentration. Since the lifespan of the erythrocytes is around 120 days, HbA1c represents the average blood sugar levels during an 8–12-week period [19]. Therefore, HbA1c is a valuable indicator for monitoring long-term average glucose control [42] and was officially adopted as one of the diagnostic criteria for diabetes by the American Diabetes Association (ADA) in 2010 [43].

It is widely recognized that HbA1c levels are related to the risk of long-term diabetic complications [44, 45]. Particularly, numerous studies have explored the association between HbA1c and lower limb ulcers in diabetes. As reported by Hsiao et al. and Pastore et al., for patients with T2D, there was an independent correlation between HbA1c variations and a long-term risk of major adverse limb events (MALEs), involving DFU and LEA [46, 47]. In addition, Eckert et al. showed that high HbA1c levels were closely related to DFU in T1D [48]. LEA is the most devastating and fearful outcome of DFU, and some studies also pointed out that high HbA1c is an independent risk factor for LEA in DFU patients [49, 50]. Conversely, a number of other studies showed no significant difference in HbA1c levels between amputees and nonamputees in DFU patients [13, 51, 52]. In light of these contradictory reports, more investigation is necessary to fully characterize these findings.

Different HbA1c levels may have different effects. For nonpregnant individuals, the ADA defined prediabetes as having an HbA1c range of 5.7%–6.4% and HbA1c ≥ 6.5% to diagnose diabetes [53]. Most international guidelines, such as the ADA and the European Association for the Study of Diabetes (EASD), recommended an HbA1c goal of 7% or lower for glycemic control [54]. DFU patients had poor glycemic control with elevated HbA1c levels, usually higher than 9% [55]. One meta-analysis suggested that an HbA1c range of 7.0%–7.7% could significantly reduce the risk of microvascular and macrovascular complications in T2D [56]. Besides, a hospital-based case–control study found that HbA1c level ≥ 8.0% was a risk factor for LEA in DFU patients [57]. Accordingly, our results indicated a strong correlation between HbA1c levels and the incidence of DFUs, and elevated HbA1c levels were associated with a higher risk of DFUs. We categorized the participants into six groups based on HbA1c levels: ≤ 42, 42–53, 53–64, 64–75, 75–86, and > 86 mmol/mol (equivalent to ≤ 6%, 6%–7%, 7%–8%, 8%–9%, 9%–10%, and > 10%). Consequently, there was a positive correlation between the incidence of lower limb ulcers and HbA1c levels when the latter was greater than 7%. However, the risk of lower limb ulcers was not further reduced by lowering the HbA1c level when it was below 53 mmol/mol. Furthermore, subgroup and sensitivity analyses produced the same results in different subgroups, based on several potential risk factors, including sex, age, BMI, smoking status, physical activity, and duration of diabetes. Thus, we concluded that the optimal target range of HbA1c levels for preventing lower limb ulcers in diabetes was less than 53 mmol/mol.

The subgroup analyses highlighted similarities and differences in the relationship between HbA1c levels and lower limb ulcers in different subgroups. For instance, men are more susceptible to elevated HbA1c levels. These findings provide valuable insights into targeted prevention strategies in clinical practice. Clinicians should emphasize the importance of blood glucose control in diabetes patients, particularly in those with a higher susceptibility to elevated HbA1c, such as men or individuals with a long history of diabetes.

A positive but not significant association between genetically proxied HbA1c and the risk of lower limb ulcers was observed in both linear and nonlinear MR analyses, which was consistent with the positive associations in the observational analyses. A previous study showed a linear causal relationship between HbA1c levels and the risk of coronary heart disease [58]. We think that the reason why MR analyses were not significant in our study was due to the limited sample size, which limits the statistical power of MR analyses. In the future, MR analysis with larger sample sizes and greater statistical power is still needed to be conducted to elucidate the potential causal relationship between HbA1c and lower limb ulcer risk. Linear MR analysis indicated that for every 1-mmol/mol reduction in HbA1c, the risk of lower limb ulcers in diabetic patients decreased by 9%, although the *p* value did not reach statistical significance. However, by integrating the results of observational studies, we emphasize the critical role of blood glucose in the risk of lower limb ulcers. Clinicians should focus on diabetic patients with high HbA1c levels, as they are at elevated risk of lower limb ulcers. These high-risk patients require intensive blood glucose management to reduce their blood glucose levels, thereby decreasing the risk of future lower limb ulcers.

Our findings emphasize the importance of controlling blood glucose levels to prevent lower limb ulcers, which is consistent with the findings of many previous studies. These insights can aid clinicians in making informed decisions and provide valuable information for developing personalized strategies to prevent lower limb ulcers in diabetes patients.

This study had some limitations. First, although the *F* statistic of the PRS for HbA1c was greater than 10, the *R*^2^ between them was only 0.3%, which also limited the statistical power of the MR analysis. Therefore, the results of the MR analysis served only as supplementary evidence. Second, in sex-specific MR analyses, there was a noticeable difference in the results between men and women. This discrepancy may be due to differences in sample size, lifestyle, and the association between PRS and HbA1c levels. Third, lower limb ulcer events were defined by *ICD-10* codes, which may have resulted in misclassification of the case status. While official records ensured the specificity of the *ICD-10* codes, there was still a possibility that some cases might have been misclassified as controls.

## 5. Conclusion

In summary, we found that high HbA1c levels were associated with a higher risk of lower limb ulcers in diabetic patients in observational analyses, although MR analyses provided a positive but not significant association between genetically proxied HbA1c and lower limb ulcer risk. Moreover, subgroup and sensitivity analyses also revealed a positive correlation between HbA1c levels and the risk of lower limb ulcers. Besides, our results recommended an HbA1c goal of < 53 mmol/mol to decrease the incidence of diabetic ulcers. Future studies should concentrate on whether prevention and treatment strategies for lower limb ulcers can be developed based on HbA1c levels for diabetic patients.

## Figures and Tables

**Table 1:** Comparisons of baseline characteristics between the healthy and lower limb ulcer cases.

**Characteristics**	**Total participants (** **n** = 23,434**)**	**Control (** **n** = 22,333**)**	**Incidence (** **n** = 1101**)**	**p**
Age, years	61 (55, 65)	61 (55, 65)	62 (56, 66)	< 0.001
< 60	9310 (39.7)	8922 (39.9)	388 (35.2)	
≥ 60	14,124 (60.3)	13,411 (60.1)	713 (64.8)	
Sex				< 0.001
Female	8750 (37.3)	8449 (37.8)	301 (27.3)	
Male	14,684 (62.7)	13,884 (62.2)	800 (72.7)	
BMI, kg/m^2^				< 0.001
< 25	2631 (11.2)	2546 (11.4)	85 (7.7)	
25–29.9	7999 (34.1)	7736 (34.6)	263 (23.9)	
≥ 30	12,616 (53.8)	11,888 (53.2)	728 (66.1)	
Missing	188 (0.8)	163 (0.7)	25 (2.3)	
Smoking status				< 0.001
Never	10,512 (44.9)	10,094 (45.2)	418 (38.0)	
Previous	10,126 (43.2)	9623 (43.1)	503 (45.7)	
Current	2552 (10.9)	2382 (10.7)	170 (15.4)	
Missing	244 (1.0)	234 (1.0)	10 (0.9)	
Drinking status				0.001
Never or special occasions	8089 (34.5)	7655 (34.3)	434 (39.4)	
1–3 times/month	2830 (12.1)	2689 (12.0)	141 (12.8)	
1–2 times/week	5415 (23.1)	5192 (23.2)	223 (20.3)	
3–4 times/week	3590 (15.3)	3453 (15.5)	137 (12.4)	
Daily or almost daily	3404 (14.5)	3245 (14.5)	159 (14.4)	
Missing	106 (0.5)	99 (0.4)	7 (0.6)	
Physical activity				< 0.001
Never	3273 (14.0)	3036 (13.6)	237 (21.5)	
Low	1383 (5.9)	1285 (5.8)	98 (8.9)	
Medium	17,469 (74.5)	16,783 (75.1)	686 (62.3)	
High	867 (3.7)	839 (3.8)	28 (2.5)	
Missing	442 (1.9)	390 (1.7)	52 (4.7)	
Townsend	−1.2 (−3.2, 2.1)	−1.3 (−3.2, 2)	0 (−2.7, 3.4)	< 0.001
Ethnicity				< 0.001
Other	5221 (22.3)	5022 (22.5)	199 (18.1)	
Caucasian	18,213 (77.7)	17,311 (77.5)	902 (81.9)	
Duration of diabetes, years				< 0.001
≤ 3	7006 (29.9)	6836 (30.6)	170 (15.4)	
3–10	9927 (42.4)	9505 (42.6)	422 (38.3)	
> 10	6501 (27.7)	5992 (26.8)	509 (46.2)	
Diabetic medication				< 0.001
No insulin or pills	6254 (26.7)	6134 (27.5)	120 (10.9)	
Only diabetes pills	12,093 (51.6)	11,562 (51.8)	531 (48.2)	
Insulin and/or others	5087 (21.7)	4637 (20.8)	450 (40.9)	
HbA1c, mmol/mol	50.5 (43.7, 59.6)	50.2 (43.6, 59.1)	57.4 (48.1, 71.2)	< 0.001
Glucose, mmol/L	6.5 (5.3, 8.9)	6.5 (5.3, 8.8)	7.7 (5.6, 11.1)	< 0.001
HDL, mmol/L	1.1 (1.0, 1.4)	1.1 (1.0, 1.4)	1.1 (0.9, 1.3)	< 0.001
LDL, mmol/L	2.6 (2.2, 3.1)	2.6 (2.2, 3.1)	2.5 (2.1, 3.0)	0.087
TC, mmol/L	4.4 (3.8, 5.0)	4.4 (3.8, 5.0)	4.3 (3.7, 4.9)	0.016
TG, mmol/L	1.8 (1.3, 2.6)	1.8 (1.3, 2.6)	2.0 (1.4, 2.9)	< 0.001
CRP, mg/L	1.8 (0.9, 3.8)	1.8 (0.9, 3.8)	2.7 (1.2, 5.7)	< 0.001

*Note:* The baseline characteristics of participants were described as *n* (%) for categorical variables, or median (interquartile range, IQR) for continuous variables. *p* values of continuous variables were estimated by the Mann–Whitney *U* test, and those of categorical variables were estimated by the chi-square test.

**Table 2:** Associations of HbA1c with lower limb ulcers among patients with diabetes.

**HbA1c (mmol/mol)**	**Case/** **N**	**Incidence density/1000-person years**	**HR (95% CI)**		
			**Model 1**	**Model 2**	**Model 3**
Per 5.5 mmol/mol	1101/23,434	3.79	1.19 (1.18–1.22)	1.19 (1.17–1.21)	1.17 (1.14–1.19)
≤ 42	127/4572	2.22	0.83 (0.67–1.02)	0.85 (0.69–1.05)	0.93 (0.76–1.15)
42–53	308/9159	2.68	Ref	Ref	Ref
53–64	262/5590	3.77	1.40 (1.19–1.66)	1.42 (1.20–1.67)	1.24 (1.05–1.46)
64–75	187/2424	6.34	2.38 (1.99–2.86)	2.40 (2.00–2.88)	1.98 (1.65–2.39)
75–86	103/963	8.95	3.38 (2.71–4.23)	3.29 (2.63–4.13)	2.68 (2.13–3.37)
> 86	114/726	14.03	5.39 (4.35–6.68)	5.43 (4.36–6.77)	4.52 (3.62–5.65)
*p* for trend	—	—	< 0.001	< 0.001	< 0.001

*Note:* Model 1 was not adjusted for any covariates. Model 2 was adjusted for age, sex, BMI, Townsend deprivation index, ethnicity, smoking status, drinking status, physical activity, and season of blood collection. Model 3 was further adjusted for the duration of diabetes.

**Table 3:** Stratified analyses of the associations of HbA1c with the risk of lower limb ulcers in diabetes patients.

**Subgroups**	**≤ 42** **mmol/mol**	**42–53** **mmol/mol**	**53–64** **mmol/mol**	**64–75** **mmol/mol**	**75–86** **mmol/mol**	**> 86** **mmol/mol**
Sex						
Male (*n* = 14,684)						
Cases/*N*	94/2967	223/5688	185/3468	140/1502	75/625	83/434
Incidence density	2.57	3.18	4.36	7.82	10.22	17.78
HR (95% CI)	0.92 (0.72–1.18)	Ref	1.23 (1.01–1.50)	2.13 (1.72–2.65)	2.72 (2.08–3.56)	5.11 (3.94–6.64)
Female (*n* = 8750)						
Cases/*N*	33/1605	85/3471	77/2122	47/922	28/338	31/292
Incidence density	1.60	1.90	2.83	4.06	6.72	8.96
HR (95% CI)	0.95 (0.63–1.42)	Ref	1.26 (0.92–1.72)	1.63 (1.13–2.35)	2.45 (1.58–3.81)	3.21 (2.08–4.93)
Age, years						
< 60 (*n* = 9310)						
Cases/*N*	36/1795	85/3232	74/2179	76/1134	47/519	70/451
Incidence density	1.55	2.03	2.59	5.28	7.28	13.48
HR (95% CI)	0.86 (0.58–1.28)	Ref	1.08 (0.79–1.48)	1.94 (1.42–2.66)	2.48 (1.73–3.56)	4.57 (3.31–6.33)
≥ 60 (*n* = 14,124)						
Cases/*N*	91/2777	223/5927	188/3411	111/1290	56/444	44/275
Incidence density	2.68	3.06	4.58	7.35	11.09	14.99
HR (95% CI)	0.94 (0.74–1.21)	Ref	1.30 (1.07–1.58)	1.95 (1.54–2.45)	2.64 (1.96–3.56)	3.85 (2.76–5.35)
BMI, kg/m^2^						
< 30 (*n* = 10,630)						
Cases/*N*	40/2287	99/4154	89/2507	56/1038	37/386	27/258
Incidence density	1.39	1.89	2.83	4.39	7.91	9.28
HR (95% CI)	0.83 (0.58–1.21)	Ref	1.34 (1.00–1.79)	2.03 (1.45–2.84)	3.64 (2.47–5.38)	5.37 (3.45–8.34)
≥ 30 (*n* = 12,616)						
Cases/*N*	82/2251	205/4942	165/3034	129/1362	63/568	84/459
Incidence density	2.92	3.32	4.39	7.85	9.32	16.39
HR (95% CI)	0.95 (0.74–1.23)	Ref	1.15 (0.94–1.42)	1.97 (1.57–2.46)	2.26 (1.69–3.01)	4.34 (3.34–5.64)
Smoking status						
Never (*n* = 10,512)						
Cases/*N*	52/1936	99/4145	96/2535	80/1114	43/425	48/357
Incidence density	2.10	1.86	2.97	5.75	8.25	11.46
HR (95% CI)	1.36 (0.97–1.91)	Ref	1.37 (1.04–1.82)	2.51 (1.86–3.39)	3.53 (2.45–5.08)	5.47 (3.83–7.81)
Other (*n* = 12,678)						
Cases/*N*	72/2599	207/4919	165/2992	105/1286	59/519	65/363
Incidence density	2.25	3.43	4.53	6.87	9.75	16.76
HR (95% CI)	0.72 (0.55–0.94)	Ref	1.17 (0.95–1.44)	1.72 (1.35–2.18)	2.33 (1.73–3.13)	4.03 (3.02–5.39)
Physical activity						
Never or low (*n* = 4656)						
Cases/*N*	45/810	87/1710	81/1123	49/556	33/250	40/207
Incidence density	4.63	4.23	6.01	7.54	11.59	18.56
HR (95% CI)	1.17 (0.82–1.69)	Ref	1.30 (0.95–1.76)	1.53 (1.07–2.19)	2.42 (1.61–3.64)	4.61 (3.12–6.81)
Medium or high (*n* = 18,336)						
Cases/*N*	74/3685	208/7289	169/4367	129/1819	62/682	69/494
Incidence density	1.59	2.25	3.07	5.73	7.78	12.02
HR (95% CI)	0.82 (0.63–1.07)	Ref	1.21 (0.98–1.48)	2.25 (1.80–2.81)	2.98 (2.24–3.96)	4.83 (3.65–6.41)
Diabetes duration, years						
≤ 3 (*n* = 7006)						
Cases/*N*	36/2134	68/3097	30/1130	18/351	7/161	11/133
Incidence density	1.32	1.73	2.10	4.06	3.44	6.80
HR (95% CI)	0.80 (0.53–1.21)	Ref	1.15 (0.75–1.77)	2.35 (1.39–3.98)	1.87 (0.85–4.12)	3.95 (2.06–7.59)
> 3 (*n* = 16,428)						
Cases/*N*	91/2438	240/6062	232/4460	169/2073	96/802	103/593
Incidence density	3.04	3.18	4.20	6.75	10.14	15.82
HR (95% CI)	0.96 (0.76–1.23)	Ref	1.35 (1.13–1.62)	2.20 (1.80–2.68)	3.17 (2.49–4.03)	5.17 (4.08–6.56)

*Note:* Models were adjusted for age, sex, BMI, Townsend deprivation index, ethnicity, smoking status, drinking status, physical activity, season of blood collection, and the duration of diabetes, except for the stratifying factors. Some participants had missing data of stratifying factors including BMI, smoking status, and physical activity.

## Data Availability

The data that support the findings of this study are available from the UK Biobank study, but restrictions apply to the availability of these data, which were used under license for the current study and therefore are not publicly available. Data are however available from the authors upon reasonable request and with the permission of the UK Biobank.

## Supporting Information

Supporting Information Additional supporting information can be found online in the Supporting Information section. Table S1 summarizes the baseline characteristics according to HbA1c concentration. Table S2 shows the multiplicative interaction between the grouped variables and HbA1c level. Tables S3–S7 show the results of sensitivity analyses. Table S8 shows the stratification information of the total population determined by the doubly ranked method. Figure S1 shows the association between the genetic instrument and HbA1c as well as a series of risk factors. Figure S2 shows the nonlinear MR estimated fitted by the doubly ranked method for the total population. Figure S3 shows the nonlinear MR estimated for HbA1c on the risk of lower limb ulcers in sex-stratified analyses.
