# Supplementary material for: HbA1c and the Risk of Lower Limb Ulcers Among Diabetic Patients: An Observational and Genetics Study
Source: J Diabetes Res. 2025 Mar 29;2025:4744194. doi: 10.1155/jdr/4744194 (PMC11972128; doi:10.1155/jdr/4744194)
Supplement: Supporting Information — Additional supporting information can be found online in the Supporting Information section. Table S1 summarizes the baseline characteristics according to HbA1c concentration. Table S2 shows the multiplicative interaction between the grouped variables and HbA1c level. Tables S3–S7 show the results of sensitivity analyses. Table S8 shows the stratification information of the total population determined by the doubly ranked method. Figure S1 shows the association between the genetic instrument and HbA1c as well as a series of risk factors. Figure S2 shows the nonlinear MR estimated fitted by the doubly ranked method for the total population. Figure S3 shows the nonlinear MR estimated for HbA1c on the risk of lower limb ulcers in sex-stratified analyses. [file 4744194.f1.docx]

**HbA1c and the risk of lower limb ulcers among diabetic patients: an observational and genetics study**

Guojun Guo^1*^, Yunlong Guan^2*^, Yuhuan Chen^2*^, Yuge Ye^2^, Zeyu Gan^2, 3^, Xi Cao^2^, Zhenbing Chen^1#^, Xingjie Hao^2#^

^1^Department of Hand Surgery, Union Hospital, Tongji Medical College, Huazhong University of Science and Technology, Wuhan 430022, China

^2^Department of Epidemiology and Biostatistics, School of Public Health, Tongji Medical College, Huazhong University of Science and Technology, 13 Hangkong Road, Wuhan, Hubei, 430030, China

^3^School of Medicine and Health Management, Tongji Medical College, Huazhong University of Science and Technology, 13 Hangkong Road, 430030, Wuhan, Hubei, China

^*^These authors contributed equally: Guojun Guo, Yunlong Guan and Yuhuan Chen.

^#^Correspondence should be addressed to Xingjie Hao <xingjie@hust.edu.cn> or Zhenbing Chen < zbchen@hust.edu.cn >.

**Table S1.** Baseline characteristics of participants with diabetes according to HbA1c concentrations.

**Table S2** The multiplicative interaction between the grouped variables and HbA1c level.

**Table S3.** Associations of HbA1c with the risk of lower limb ulcers among diabetes patients with complete covariates.

**Table S4.** Associations of HbA1c with the risk of lower limb ulcers among type 2 diabetes patients.

**Table S5.** Associations of HbA1c with the risk of lower limb ulcers among diabetes patients further adjusted for diabetic medication use.

**Table S6.** Associations of HbA1c with the risk of lower limb ulcers among diabetes patients after excluding participants with events in the first two years of follow-up.

**Table S7.** Associations of HbA1c with the risk of lower limb ulcers using inverse probability weighting.

**Table S8.** Stratification of the total population determined by the doubly ranked method.

**Figure S1.** The association between genetic instrument and HbA1c as well as a series of risk factors.

**Figure S2.** Non-linear MR estimated fitted by the doubly ranked method with 53 mmol/mol as the reference for total population.

**Figure S3.** Non-linear MR estimated for HbA1c on the risk of lower limb ulcers in sex-stratified analyses.

Table S1. Baseline characteristics of participants with diabetes according to HbA1c levels.

| Characteristics | HbA1c concentrations, mmol/mol | | | | | |
| --- | --- | --- | --- | --- | --- | --- |
|  | ≤42 | 42-53 | 53-64 | 64-75 | 75-86 | >86 |
| **Participants, n** | 4,572 | 9,159 | 5,590 | 2,424 | 963 | 726 |
| **Age, years** | 61 (56, 65) | 62 (57, 66) | 61 (56, 65) | 60 (54, 65) | 59 (52, 63) | 57 (51, 62) |
| <60 | 1,795 (39.3) | 3,232 (35.3) | 2,179 (39.0) | 1,134 (46.8) | 519 (53.9) | 451 (53.9) |
| ≥60 | 2,777 (60.7) | 5,927 (64.7) | 3,411 (61.0) | 1,290 (53.2) | 444 (46.1) | 275 (46.1) |
| **Sex** |  |  |  |  |  |  |
| Female | 1,605 (35.1) | 3,471 (37.9) | 2,122 (38.0) | 922 (38.0) | 338 (35.1) | 292 (35.1) |
| Male | 2,967 (64.9) | 5,688 (62.1) | 3,468 (62.0) | 1,502 (62.0) | 625 (64.9) | 434 (64.9) |
| **BMI, kg/m^2^** |  |  |  |  |  |  |
| <25 | 601 (13.1) | 968 (10.6) | 629 (11.3) | 264 (10.9) | 98 (10.2) | 71 (10.2) |
| 25-29.9 | 1,686 (36.9) | 3,186 (34.8) | 1,878 (33.6) | 774 (31.9) | 288 (29.9) | 187 (29.9) |
| ≥30 | 2,251 (49.2) | 4,942 (54.0) | 3,034 (54.3) | 1,362 (56.2) | 568 (59.0) | 459 (59.0) |
| Missing | 34 (0.7) | 63 (0.7) | 49 (0.9) | 24 (1.0) | 9 (0.9) | 9 (0.9) |
| **Smoking status** |  |  |  |  |  |  |
| Never | 1,936 (42.3) | 4,145 (45.3) | 2,535 (45.3) | 1,114 (46.0) | 425 (44.1) | 357 (44.1) |
| Previous | 2,134 (46.7) | 3,951 (43.1) | 2,404 (43.0) | 991 (40.9) | 388 (40.3) | 258 (40.3) |
| Current | 465 (10.2) | 968 (10.6) | 588 (10.5) | 295 (12.2) | 131 (13.6) | 105 (13.6) |
| Missing | 37 (0.8) | 95 (1.0) | 63 (1.1) | 24 (1.0) | 19 (2.0) | 6 (2.0) |
| **Drinking status** |  |  |  |  |  |  |
| Never or special occasions | 1,384 (30.3) | 3,078 (33.6) | 1,993 (35.7) | 933 (38.5) | 369 (38.3) | 332 (38.3) |
| 1-3 times/month | 473 (10.3) | 1,095 (12.0) | 671 (12.0) | 335 (13.8) | 135 (14.0) | 121 (14.0) |
| 1-2 times/week | 1,034 (22.6) | 2,162 (23.6) | 1,318 (23.6) | 521 (21.5) | 223 (23.2) | 157 (23.2) |
| 3-4 times/week | 816 (17.8) | 1,458 (15.9) | 808 (14.5) | 336 (13.9) | 113 (11.7) | 59 (11.7) |
| Daily or almost daily | 845 (18.5) | 1,332 (14.5) | 776 (13.9) | 290 (12.0) | 110 (11.4) | 51 (11.4) |
| Missing | 20 (0.4) | 34 (0.4) | 24 (0.4) | 9 (0.4) | 13 (1.3) | 6 (1.3) |
| **Physical activity** |  |  |  |  |  |  |
| Never | 560 (12.2) | 1,210 (13.2) | 768 (13.7) | 401 (16.5) | 183 (19,0) | 151 (19.0) |
| Low | 250 (5.5) | 500 (5.5) | 355 (6.4) | 155 (6.4) | 67 (7,0) | 56 (7.0) |
| Medium | 3,466 (75.8) | 6,980 (76.2) | 4,150 (74.2) | 1,745 (72.0) | 652 (67.7) | 476 (67.7) |
| High | 219 (4.8) | 309 (3.4) | 217 (3.9) | 74 (3.1) | 30 (3.1) | 18 (3.1) |
| Missing | 77 (1.7) | 160 (1.7) | 100 (1.8) | 49 (2) | 31 (3.2) | 25 (3.2) |
| **Townsend** | -1.3 (-3.2, 2) | -1.3 (-3.2, 1.9) | -1.4 (-3.2, 1.9) | -1 (-3, 2.3) | -0.4 (-2.8, 3.1) | 0.5 (-2.3, 3.4) |
| **Ethnicity** |  |  |  |  |  |  |
| Other | 912 (19.9) | 2,025 (22.1) | 1,247 (22.3) | 563 (23.2) | 243 (25.2) | 231 (25.2) |
| Caucasian | 3,660 (80.1) | 7,134 (77.9) | 4,343 (77.7) | 1,861 (76.8) | 720 (74.8) | 495 (74.8) |
| **Duration of diabetes, years** |  |  |  |  |  |  |
| ≤3 | 2,134 (46.7) | 3,097 (33.8) | 1,130 (20.2) | 351 (14.5) | 161 (16.7) | 133 (16.7) |
| 3-10 | 1,779 (38.9) | 4,140 (45.2) | 2,393 (42.8) | 980 (40.4) | 356 (37.0) | 279 (37.0) |
| >10 | 659 (14.4) | 1922 (21.0) | 2067 (37.0) | 1,093 (45.1) | 446 (46.3) | 314 (46.3) |
| **Diabetic medication** |  |  |  |  |  |  |
| No insulin or pills | 2,528 (55.3) | 2,741 (29.9) | 705 (12.6) | 163 (6.7) | 64 (6.6) | 53 (6.6) |
| Only diabetes pills | 1,670 (36.5) | 5,288 (57.7) | 3,212 (57.5) | 1,168 (48.2) | 437 (45.4) | 318 (45.4) |
| Insulin and/or others | 374 (8.2) | 1,130 (12.3) | 1,673 (29.9) | 1,093 (45.1) | 462 (48.0) | 355 (48.0) |
| **Glucose, mmol/L** | 5.3 (4.8, 6.0) | 6.1 (5.2, 7.4) | 7.7 (6.2, 9.9) | 9.7 (7.3, 12.5) | 11.7 (9, 14.7) | 14.8 (11.6, 18) |
| **HDL, mmol/L** | 1.2 (1.0, 1.4) | 1.1 (1.0, 1.4) | 1.1 (1.0, 1.3) | 1.1 (0.9, 1.4) | 1.1 (0.9, 1.3) | 1.1 (0.9, 1.3) |
| **LDL, mmol/L** | 2.6 (2.2, 3.2) | 2.5 (2.1, 3.0) | 2.5 (2.1, 3.0) | 2.6 (2.2, 3.1) | 2.7 (2.3, 3.2) | 2.8 (2.3, 3.4) |
| **TC, mmol/L** | 4.4 (3.8, 5.2) | 4.3 (3.8, 5.0) | 4.3 (3.7, 5.0) | 4.3 (3.8, 5.1) | 4.5 (3.9, 5.2) | 4.7 (4.0, 5.5) |
| **TG, mmol/L** | 1.7 (1.2, 2.3) | 1.8 (1.3, 2.6) | 1.9 (1.3, 2.7) | 1.9 (1.2, 2.9) | 2.0 (1.4, 2.9) | 2.2 (1.5, 3.2) |
| **CRP, mg/L** | 1.5 (0.8, 3.1) | 1.7 (0.8, 3.5) | 1.9 (0.9, 3.9) | 2.3 (1.1, 4.8) | 2.8 (1.3, 5.4) | 3.2 (1.6, 6.1) |

The baseline characteristics of participants were described as n (%) for categorical variables, or median (interquartile range, IQR) for continuous variables.

Table S2 The multiplicative interaction between the grouped variables and HbA1c level.

| Characteristics | *P* for interaction |
| --- | --- |
| Sex | 0.581 |
| Age | 0.585 |
| BMI | 0.432 |
| Smoking status | 0.064 |
| Physical activity | 0.280 |
| Diabetes duration | 0.901 |

Table S3. Associations of HbA1c with the risk of lower limb ulcers among diabetes patients with complete covariates.

| HbA1c (mmol/mol) | Case/N | Incidence density | HR (95% CI) | | |
| --- | --- | --- | --- | --- | --- |
|  |  |  | Model 1 | Model 2 | Model 3 |
| *per* 5.5 mmol/mol | 1,022/22,652 | 3.62 | 1.20 (1.18-1.22) | 1.20 (1.18-1.22) | 1.18 (1.15-1.20) |
| ≤42 | 113/4,435 | 2.03 | 0.78 (0.63-0.97) | 0.81 (0.65-1.01) | 0.89 (0.71-1.11) |
| 42-53 | 290/8,875 | 2.60 | ref | ref | ref |
| 53-64 | 242/5,400 | 3.59 | 1.38 (1.17-1.64) | 1.40 (1.18-1.66) | 1.22 (1.03-1.45) |
| 64-75 | 176/2,337 | 6.17 | 2.39 (1.98-2.88) | 2.42 (2.01-2.92) | 2.00 (1.65-2.42) |
| 75-86 | 96/915 | 8.71 | 3.40 (2.70-4.28) | 3.38 (2.67-4.26) | 2.76 (2.18-3.49) |
| >86 | 105/690 | 13.49 | 5.35 (4.28-6.69) | 5.55 (4.42-6.97) | 4.65 (3.69-5.86) |
| *P* for trend |  |  | <0.001 | <0.001 | <0.001 |

Model 1 was not adjusted for any covariates. Model 2 was adjusted for age, sex, BMI, Townsend deprivation index, ethnicity, smoking status, drinking status, physical activity, and season of blood collection. Model 3 was further adjusted for the duration of diabetes.

Table S4. Associations of HbA1c with the risk of lower limb ulcers among type 2 diabetes patients.

| HbA1c (mmol/mol) | Case/N | Incidence density | HR (95% CI) | | |
| --- | --- | --- | --- | --- | --- |
|  |  |  | Model 1 | Model 2 | Model 3 |
| *per* 5.5 mmol/mol | 958/21,883 | 3.52 | 1.18 (1.16-1.20) | 1.18 (1.15-1.20) | 1.15 (1.13-1.18) |
| ≤42 | 125/4,528 | 2.21 | 0.83 (0.68-1.03) | 0.87 (0.71-1.08) | 0.94 (0.76-1.16) |
| 42-53 | 294/8,869 | 2.65 | ref | ref | ref |
| 53-64 | 220/5,010 | 3.53 | 1.33 (1.12-1.59) | 1.30 (1.09-1.55) | 1.18 (0.99-1.40) |
| 64-75 | 145/2,029 | 5.88 | 2.24 (1.84-2.73) | 2.12 (1.74-2.59) | 1.85 (1.51-2.26) |
| 75-86 | 84/812 | 8.61 | 3.30 (2.59-4.20) | 3.04 (2.38-3.89) | 2.62 (2.05-3.36) |
| >86 | 90/635 | 12.42 | 4.84 (3.82-6.13) | 4.75 (3.73-6.04) | 4.11 (3.22-5.25) |
| *P* for trend |  |  | <0.001 | <0.001 | <0.001 |

Model 1 was not adjusted for any covariates. Model 2 was adjusted for age, sex, BMI, Townsend deprivation index, ethnicity, smoking status, drinking status, physical activity, and season of blood collection. Model 3 was further adjusted for the duration of diabetes.

Table S5. Associations of HbA1c with the risk of lower limb ulcers among diabetes patients further adjusted for diabetic medication use.

| HbA1c (mmol/mol) | Case/N | Incidence density/  1000-person years | HR (95% CI) | | | |
| --- | --- | --- | --- | --- | --- | --- |
|  |  |  | Model 1 | Model 2 | Model 3 |  |
| *per* 5.5 mmol/mol | 1,101/23,434 | 3.79 | 1.19 (1.18-1.22) | 1.19 (1.17-1.21) | 1.17 (1.14-1.19) |  |
| ≤42 | 127/4,572 | 2.22 | 0.83 (0.67-1.02) | 0.85 (0.69-1.05) | 1.04 (0.82-1.33) |  |
| 42-53 | 308/9,159 | 2.68 | ref | ref | ref |  |
| 53-64 | 262/5,590 | 3.77 | 1.40 (1.19-1.66) | 1.42 (1.20-1.67) | 1.07 (0.88-1.29) |  |
| 64-75 | 187/2,424 | 6.34 | 2.38 (1.99-2.86) | 2.40 (2.00-2.88) | 1.59 (1.28-1.97) |  |
| 75-86 | 103/963 | 8.95 | 3.38 (2.71-4.23) | 3.29 (2.63-4.13) | 2.30 (1.77-2.98) |  |
| >86 | 114/726 | 14.03 | 5.39 (4.35-6.68) | 5.43 (4.36-6.77) | 3.41 (2.62-4.44) |  |
| *P* for trend | - | - | <0.001 | <0.001 | <0.001 |  |

Model 1 was not adjusted for any covariates. Model 2 was adjusted for age, sex, BMI, Townsend deprivation index, ethnicity, smoking status, drinking status, physical activity, and season of blood collection. Model 3 was further adjusted for the duration of diabetes and diabetic medication use.

Table S6. Associations of HbA1c with the risk of lower limb ulcers among diabetes patients after excluding participants with events in the first two years of follow-up.

| HbA1c (mmol/mol) | Case/N | Incidence density | HR (95% CI) | | |
| --- | --- | --- | --- | --- | --- |
|  |  |  | Model 1 | Model 2 | Model 3 |
| *per* 5.5 mmol/mol | 1,016/23,349 | 3.50 | 1.20 (1.18-1.22) | 1.20 (1.17-1.22) | 1.17 (1.15-1.20) |
| ≤53 | 120/4,565 | 2.10 | 0.88 (0.71-1.09) | 0.90 (0.73-1.12) | 0.99 (0.80-1.23) |
| 42-53 | 275/9,126 | 2.40 | ref | ref | ref |
| 53-64 | 242/5,570 | 3.48 | 1.45 (1.22-1.73) | 1.47 (1.24-1.75) | 1.28 (1.08-1.53) |
| 64-75 | 177/2,414 | 6.01 | 2.53 (2.10-3.06) | 2.57 (2.13-3.11) | 2.12 (1.75-2.57) |
| 75-86 | 98/958 | 8.52 | 3.62 (2.88-4.56) | 3.57 (2.83-4.50) | 2.90 (2.29-3.67) |
| >86 | 104/716 | 12.82 | 5.57 (4.45-6.98) | 5.70 (4.53-7.18) | 4.75 (3.76-6.00) |
| *P* for trend |  |  | <0.001 | <0.001 | <0.001 |

Model 1 was not adjusted for any covariates. Model 2 was adjusted for age, sex, BMI, Townsend deprivation index, ethnicity, smoking status, drinking status, physical activity, and season of blood collection. Model 3 was further adjusted for the duration of diabetes.

Table S7. Associations of HbA1c with the risk of lower limb ulcers using inverse probability weighting.

| HbA1c (mmol/mol) | Case/N | Incidence density | HR (95% CI) |
| --- | --- | --- | --- |
| ≤42 | 127/4,572 | 2.22 | 0.98 (0.79-1.22) |
| 42-53 | 308/9.159 | 2.68 | ref |
| 53-64 | 262/5,590 | 3.77 | 1.24 (1.05-1.47) |
| 64-75 | 187/2,424 | 6.34 | 2.00 (1.65-2.43) |
| 75-86 | 103/963 | 8.95 | 2.69 (2.11-3.42) |
| >86 | 114/726 | 14.03 | 4.21 (3.30-5.37) |

The HbA1c groups were weighted using inverse probability weighting for age, sex, BMI, Townsend deprivation index, ethnicity, smoking status, drinking status, physical activity, season of blood collection, and the duration of diabetes. Model was adjusted for age, sex, BMI, Townsend deprivation index, ethnicity, smoking status, drinking status, physical activity, season of blood collection, and the duration of diabetes.

Table S8. Stratification of the total population determined by the doubly ranked method.

| Stratum | *β*x | *β*y | se (*β*x) | se (*β*y) | X_mean_ | X_min_ | X_max_ |
| --- | --- | --- | --- | --- | --- | --- | --- |
| 1 | **0.70528331** | -0.01650421 | 0.09485436 | 0.14126831 | 37.0064053 | 33.6 | 42.1 |
| 2 | **0.82626598** | -0.03163355 | 0.08405178 | 0.13467778 | 41.0317504 | 36.3 | 45.9 |
| 3 | **0.81745852** | 0.14928525 | 0.08498643 | 0.1188599 | 43.8711761 | 39.1 | 48.9 |
| 4 | **0.83894408** | 0.05763375 | 0.09192419 | 0.12804417 | 46.5654335 | 41.6 | 51.9 |
| 5 | **0.70230592** | 0.04178712 | 0.10000863 | 0.12392008 | 49.2144671 | 44 | 54.7 |
| 6 | **0.63988776** | 0.01273279 | 0.10909842 | 0.10718392 | 52.0325235 | 46.3 | 58.2 |
| 7 | **0.77521555** | 0.19069084 | 0.12637255 | 0.10386857 | 55.4513528 | 48.9 | 62.6 |
| 8 | **0.69999044** | -0.08309178 | 0.15417166 | 0.0927262 | 59.6353591 | 51.8 | 68.41 |
| 9 | **0.57715257** | 0.18494902 | 0.20530785 | 0.08631274 | 65.740442 | 55.49 | 77.3 |
| 10 | **0.68652119** | 0.0381804 | 0.32513429 | 0.069771 | 77.3073481 | 61.8 | 88.3 |


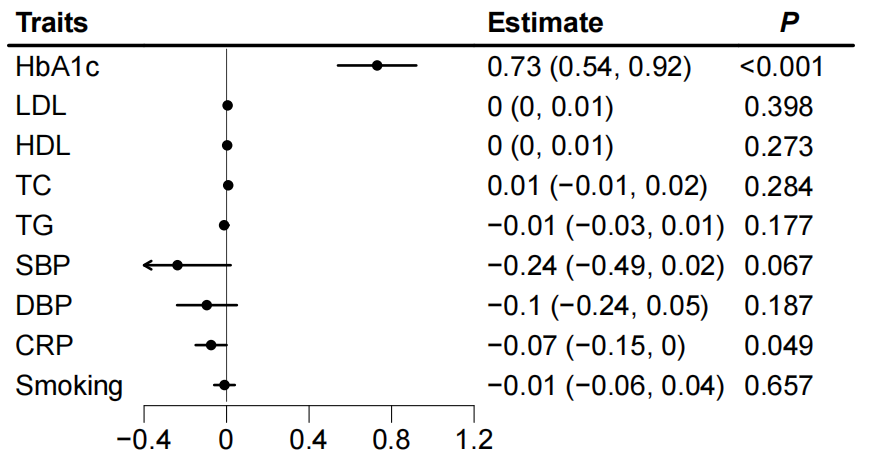


Figure S1. The association between genetic instrument (PRS_HbA1c_) and HbA1c as well as a series of risk factors.


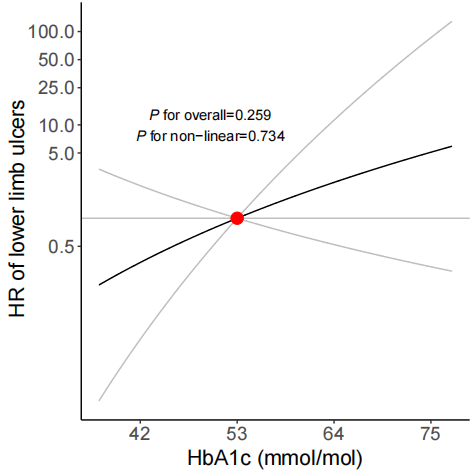


Figure S2. Non-linear MR estimated fitted by the doubly ranked method with 53 mmol/mol as the reference for total population.


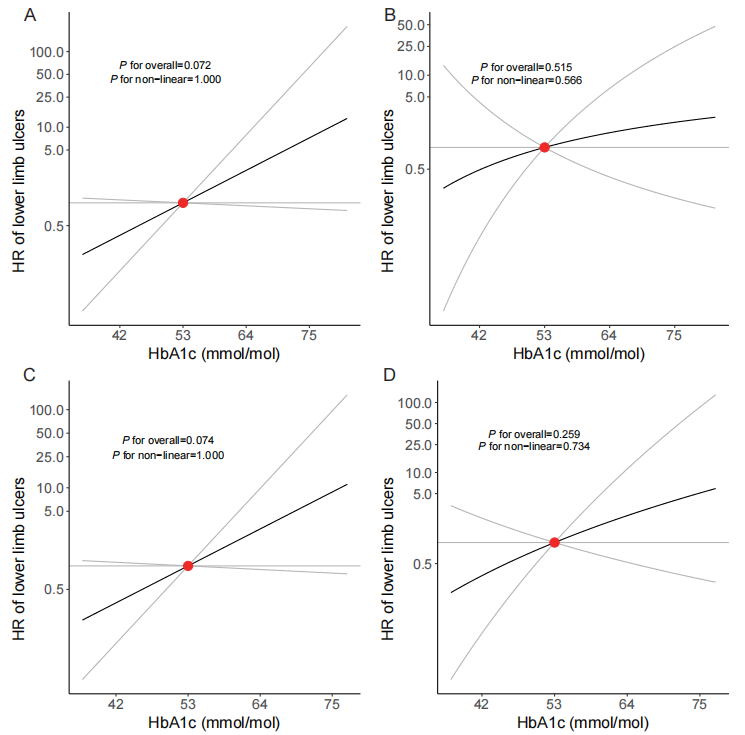


Figure S3. Non-linear MR estimated for HbA1c on the risk of lower limb ulcers in sex-stratified analyses. A. male only, the residual method; B. female only, the residual method; C. male only, the doubly ranked method; D. female only, the doubly ranked method.
